# Markedly divergent estimates of Amazon forest carbon density from ground plots and satellites

**DOI:** 10.1111/geb.12168

**Published:** 2014-04-22

**Authors:** Edward T A Mitchard, Ted R Feldpausch, Roel J W Brienen, Gabriela Lopez-Gonzalez, Abel Monteagudo, Timothy R Baker, Simon L Lewis, Jon Lloyd, Carlos A Quesada, Manuel Gloor, Hans ter Steege, Patrick Meir, Esteban Alvarez, Alejandro Araujo-Murakami, Luiz E O C Aragão, Luzmila Arroyo, Gerardo Aymard, Olaf Banki, Damien Bonal, Sandra Brown, Foster I Brown, Carlos E Cerón, Victor Chama Moscoso, Jerome Chave, James A Comiskey, Fernando Cornejo, Massiel Corrales Medina, Lola Da Costa, Flavia R C Costa, Anthony Di Fiore, Tomas F Domingues, Terry L Erwin, Todd Frederickson, Niro Higuchi, Euridice N Honorio Coronado, Tim J Killeen, William F Laurance, Carolina Levis, William E Magnusson, Beatriz S Marimon, Ben Hur Marimon Junior, Irina Mendoza Polo, Piyush Mishra, Marcelo T Nascimento, David Neill, Mario P Núñez Vargas, Walter A Palacios, Alexander Parada, Guido Pardo Molina, Marielos Peña-Claros, Nigel Pitman, Carlos A Peres, Lourens Poorter, Adriana Prieto, Hirma Ramirez-Angulo, Zorayda Restrepo Correa, Anand Roopsind, Katherine H Roucoux, Agustin Rudas, Rafael P Salomão, Juliana Schietti, Marcos Silveira, Priscila F de Souza, Marc K Steininger, Juliana Stropp, John Terborgh, Raquel Thomas, Marisol Toledo, Armando Torres-Lezama, Tinde R van Andel, Geertje M F van der Heijden, Ima C G Vieira, Simone Vieira, Emilio Vilanova-Torre, Vincent A Vos, Ophelia Wang, Charles E Zartman, Yadvinder Malhi, Oliver L Phillips

**Affiliations:** 1 School of GeoSciences, University of EdinburghEdinburgh, EH9 3JN, UK; 2 School of Geography, University of LeedsLeeds, LS2 9JT, UK; 3 Geography, College of Life and Environmental Sciences, University of ExeterExeter, EX4 4RJ, UK; 4 Jardín Botánico de MissouriOxapampa, Peru; 5 Department of Geography, University College LondonUK; 6 Faculty of Natural Sciences, Department of Life Sciences, Imperial CollegeLondon, UK; 7 Instituto Nacional de Pesquisas da AmazôniaManaus, Brazil; 8 Naturalis Biodiversity CenterLeiden, the Netherlands; 9 Institute of Environmental Biology, Utrecht UniversityUtrecht, the Netherlands; 10 Research School of Biology, Australian National UniversityCanberra, ACT, 0200, Australia; 11 Jardín Botánico de Medellín, Grupo de Investigación en Servicios Ecosistémicos y Cambio ClimáticoMedellin, Colombia; 12 Museo de Historia Natural Noel Kempff Mercado, Universidad Autónoma Gabriel René MorenoCasilla 2489, Av. Irala 565, Santa Cruz, Bolivia; 13 Remote Sensing Division, National Institute for Space Research – INPESão José dos Campos, SP, Brazil; 14 UNELLEZ-Guanare, Programa de Ciencias del Agro y el Mar, Herbario Universitario (PORT)Mesa de Cavacas, Estado Portuguesa, 3350, Venezuela; 15 IBED, University of AmsterdamPOSTBUS 94248, 1090 GE, Amsterdam, the Netherlands; 16 L'Institut National de la Recherche AgronomiqueUMR 1137 EEF, 54280, Champenoux, France; 17 Ecosystem Services Unit, Winrock InternationalArlington, VA, 22202, USA; 18 Woods Hole Research CenterFalmouth, MA, USA; 19 Universidade Federal do Acre, Centro de Ciências Biológicas e da NaturezaRio Branco, AC, 69910-900, Brazil; 20 Herbario Alfredo Paredes (QAP), Universidad Central del EcuadorQuito, Ecuador; 21 Université Paul Sabatier, Laboratoire EDBbâtiment 4R3, 31062, Toulouse, France; 22 National Park ServiceFredericksburg, VA, USA; 23 Universidad Nacional Agraria La Molina, Facultad de Ciencias ForestalesLima, Peru; 24 Universidad Nacional de San Agustín de ArequipaArequipa, Peru; 25 Geociencias, Universidade Federal de ParaBelem, Brazil; 26 Univeristy of TexasAustin, TX, USA; 27 FFCLRP-USP, Department of Biology, Universidade de São Paulo05508-090, Brazil; 28 Department of Entomology, Smithsonian InstitutionP.O. Box 37012, MRC 187, Washington, DC, 20013-7012, USA; 29 Ferrum CollegeFerum, Virginia, USA; 30 Instituto de Investigaciones de la Amazonía PeruanaAv. José A. Quiñones km. 2.5, Iquitos, Peru; 31 Museu Paraense Emilio GoeldiAv. Magalhães Barata, 376, São Braz, 66040-170, Belém, PA, Brazil; 32 World Wildlife Fund1250 24th Street, N.W., Washington, DC, 20037, USA; 33 Centre for Tropical Environmental and Sustainability Science (TESS), School of Marine and Tropical Biology, James Cook UniversityCairns, Queensland, 4878, Australia; 34 Universidade do Estado de Mato Grosso, Campus de Nova XavantinaCaixa Postal 08, CEP 78.690-000, Nova Xavantina, MT, Brazil; 35 Department of Civil Engineering, Indian Institute of TechnologyRoorkee, Uttarakhand, 247667, India; 36 Centro de Biociências e Biotecnologia, Universidade Estadual do Norte FlumineseCampos dos Goytacazes, RJ, Brasil; 37 Puyo, Universidad Estatal AmazónicaPaso lateral km 2½ via a Napo, Pastaza, Ecuador; 38 Universidad Nacional de San Antonio Abad del CuscoCusco, Peru; 39 Escuela de Ingeniería Forestal, Universidad Técnica del NorteEcuador; 40 Universidad Autónoma del BeniRiberalta, Beni, Bolivia; 41 Forest Ecology and Forest Management Group, Wageningen UniversityP.O. Box 47, 6700 AA, Wageningen, the Netherlands; 42 Instituto Boliviano de Investigación ForestalSanta Cruz, Bolivia; 43 Center for Tropical Conservation, Duke UniversityBox 90381, Durham, NC, 27708, USA; 44 Centre for Biodiversity Research, School of Environmental Sciences, University of East AngliaNorwich, NR4 7JT, UK; 45 Instituto de Ciencias Naturales, Universidad Nacional de ColombiaBogota, Colombia; 46 Universidad de Los AndesMerida, Venezuela; 47 Department of Biology, University of FloridaP.O. 118526, 511 Bartram Hall, Gainesville, FL, 32611-8526, USA; 48 Universidad Nacional de ColombiaLeticia, Colombia; 49 Center for Applied Biodiversity Science, Conservation InternationalWashington, DC, USA; 50 Institute for Environment and Sustainability, Joint Research Centre of the European CommissionVia Enrico Fermi, 2748 TP 440, I-21027, Ispra, Italy; 51 Nicholas School of the Environment, Duke UniversityBox 90381, Durham, NC, 27708, USA; 52 Iwokrama International Centre77 High Street Kingston, Georgetown, Guyana; 53 Universidad Autónoma Gabriel René MorenoSanta Cruz, Bolivia; 54 University of Wisconsin-MilwaukeeP.O Box 413, Milwaukee, WI, 53201, USA; 55 Smithsonian Tropical Research InstituteApartado, Postal 0843-03092, Panamá, Panama; 56 Núcleo de Estudos e Pesquisas Ambientais, Universidade Estadual de CampinasCampinas, Brazil; 57 Lab of Landscape Ecology and Conservation Biology, Northern Arizona UniversityFlagstaff, AZ, USA; 58 School of Geography and the Environment, University of OxfordOxford, UK

**Keywords:** Above-ground biomass, allometry, carbon cycle, REDD+, remote sensing, satellite mapping, wood density

## Abstract

**Aim:**

The accurate mapping of forest carbon stocks is essential for understanding the global carbon cycle, for assessing emissions from deforestation, and for rational land-use planning. Remote sensing (RS) is currently the key tool for this purpose, but RS does not estimate vegetation biomass directly, and thus may miss significant spatial variations in forest structure. We test the stated accuracy of pantropical carbon maps using a large independent field dataset.

**Location:**

Tropical forests of the Amazon basin. The permanent archive of the field plot data can be accessed at: http://dx.doi.org/10.5521/FORESTPLOTS.NET/2014_1

**Methods:**

Two recent pantropical RS maps of vegetation carbon are compared to a unique ground-plot dataset, involving tree measurements in 413 large inventory plots located in nine countries. The RS maps were compared directly to field plots, and kriging of the field data was used to allow area-based comparisons.

**Results:**

The two RS carbon maps fail to capture the main gradient in Amazon forest carbon detected using 413 ground plots, from the densely wooded tall forests of the north-east, to the light-wooded, shorter forests of the south-west. The differences between plots and RS maps far exceed the uncertainties given in these studies, with whole regions over- or under-estimated by > 25%, whereas regional uncertainties for the maps were reported to be < 5%.

**Main conclusions:**

Pantropical biomass maps are widely used by governments and by projects aiming to reduce deforestation using carbon offsets, but may have significant regional biases. Carbon-mapping techniques must be revised to account for the known ecological variation in tree wood density and allometry to create maps suitable for carbon accounting. The use of single relationships between tree canopy height and above-ground biomass inevitably yields large, spatially correlated errors. This presents a significant challenge to both the forest conservation and remote sensing communities, because neither wood density nor species assemblages can be reliably mapped from space.

## Introduction

Amazonia contains half of all remaining tropical moist forest (Fritz *et al*., 2003). The total vegetation carbon storage of Amazon basin tropical forests has been subject to a wide range of estimates (Houghton *et al*., 2001; Malhi *et al*., 2006; Saatchi *et al*., 2007). These have varied from 58 Pg C (Olson *et al*., 1983) to 134 Pg C (Fearnside, 1997, scaled to whole basin), although there is now some general consensus in the middle of this range [e.g. 93 ± 23 Pg C (Malhi *et al*., 2006), 86 ± 17 Pg C (Saatchi *et al*., 2007) and 89 Pg C (FAO, 2010)]. However, these estimates of carbon stocks mask large differences at a smaller spatial scale, as local variations are cancelled out when summing over large areas: the spatial patterns visible in different maps of above-ground biomass (AGB) vary greatly, with little consistency even between studies that use similar methods and input data (Houghton *et al*., 2001).

It is of great importance that the distribution of carbon storage across the Amazon be well-characterized. Although there are many reasons that make it desirable to protect tropical forests, the protection of their carbon stocks and potential as a future carbon sink have made their preservation a current policy priority. A major initiative in international climate negotiations, Reducing Emissions from Deforestation and forest Degradation (REDD+), envisages payments in return for forest conservation. Though REDD+ is not yet operational, voluntary-sector afforestation/reforestation and REDD+ projects already exist, with REDD+ credit sales equal to $85 million in 2010 (Diaz *et al*., 2011). Country-to-country cash transfers have also taken place, with Norway leading the way, committing US$1 billion to the government of Indonesia, a similar amount to Brazil's Amazon Fund, and $250 million to Guyana, in return for their meeting goals for reducing rates of forest loss (Caravani *et al*., 2012). Other sources of conservation and development funding also assess projects based on their carbon impact: indeed, one of the stated criteria applied to all USAID funding (equivalent to US$40 billion in 2012) is to be carbon-positive where possible (U.S. Agency for International Development, 2012).

For a wide variety of conservation and sustainable forest management projects, forest carbon stocks – and changes in these stocks – must be estimated with confidence. Accurate estimation, however, still faces major challenges: indeed, in a comparison of estimates of carbon emissions from deforestation in the Amazon, the biggest cause of discrepancies between estimates was found to be due to carbon mapping, higher than the uncertainty in the mapping of deforestation (Gutierrez-Velez & Pontius, 2012). AGB is the largest carbon pool in most tropical forests, and also tends to be the best characterized because it is relatively easy to measure, with other carbon pools often estimated as a simple ratio of AGB (GOFC-GOLD, 2009).

Biomass maps of the Amazon region have been created in a number of ways. Some have used direct extrapolations from field-plot measurements, either multiplying the total area of forest by mean biomass density values (Olson *et al*., 1983; Fearnside, 1997; FAO, 2010) or by two-dimensional kriging (Malhi *et al*., 2006); others have used environmental gradients to co-krig field-plot measurements (U.S. Agency for International Development, 2012); and others have used remote-sensing (RS) data (Saatchi *et al*., 2007). In the absence of continuous field measurements throughout an area of interest, RS datasets should provide the most accurate maps, because every location can be directly observed. Methods based solely on ground plots will only ever be able to sample a very small percentage of the total area, and due to access difficulties, a network of ground points will normally be biased towards more easily accessible regions (concentrated near rivers, roads and scientific field stations). However, using current technology AGB cannot be directly estimated from space (Woodhouse *et al*., 2012), and thus field plots remain essential for calibrating and validating RS maps. Ground-based estimates, and thereby calibrations of RS maps, are themselves limited by the small quantity of destructive biomass data available, which reduces the confidence in allometric equations used to convert ground data into estimates of AGB (Feldpausch *et al*., 2012).

Two recent maps have been published that estimate AGB across the tropics at 1 km (Saatchi *et al*., 2011; subsequently called *RS1*) and 500 m (Baccini *et al*., 2012; *RS2*) resolution, aimed specifically at providing baseline data for REDD+, with the data being widely disseminated and used. Both maps use similar methods and datasets: they take millions of discrete 0.25-ha canopy height estimates from the Ice, Cloud and Land Elevation Satellite (ICESat) Geoscience Laser Altimeter System (GLAS) LiDAR sensor, convert these to estimates of AGB using empirically derived models that relate LiDAR variables to AGB using field plots located under some GLAS footprints, and use ancillary full-coverage RS layers to extrapolate these point AGB estimates across the landscape. The ancillary RS layers are visual and infra-red spectrum optical data from the Moderate Resolution Imaging Spectroradiometer (MODIS) sensors, elevation data from the Shuttle Radar Topography Mission (SRTM), and, in the case of *RS1* only, QuikSCAT radar scatterometer data. The extrapolation of AGB is performed using multi-variable nonlinear models, MaxEnt in *RS1* and RandomForests in *RS2*. Though they use similar input data, the nominal date of the resulting AGB maps differs between the two, with *RS1* dated as ‘early 2000s’, and *RS2* 2007–2008. *RS1* provides a continuous uncertainty map, giving an uncertainty of ± 6% to ± 53% associated with every pixel, and assumes these errors are spatially uncorrelated to give an uncertainty for the total carbon stock for Amazonia of < ± 1% (Saatchi *et al*., 2011). *RS2* does not provide a pixel-level uncertainty map but instead held back some training data, using a Monte Carlo approach to estimate uncertainty at the level of Amazonia as ± 7% (Baccini *et al*., 2012).

In both cases, the primary calibration data used to produce the maps is derived from profiles of tree height from the ICESat GLAS sensor. Although these data do include some information about the structural characteristics of the forest within the LiDAR footprints, canopy height is the principal parameter detected (Lefsky *et al*., 2005). However, allometric equations that relate physical attributes of trees to their above-ground biomass normally rely on three parameters: in addition to tree height (*H*), tree diameter at 1.3 m (*D*) and wood density (ρ) are very important (Chave *et al*., 2005), and mean values and ratios between these parameters vary significantly between regions (Chave *et al*., 2005; Feldpausch *et al*., 2012; Quesada *et al*., 2012), associated with different species communities (ter Steege *et al*., 2006). We know that wood density increases from west to east across Amazonia (Baker *et al*., 2004; ter Steege *et al*., 2006), inversely correlated to stem turnover rate (Quesada *et al*., 2012). This gradient is driven by soil fertility, notably total soil phosphorus and the concentration of exchangeable potassium ions (Aragão *et al*., 2009), and especially by the physical qualities of the soil. Thus, the fertile but shallower soils of the western Amazon lead to higher productivity and faster turnover, and a set of species with low wood density; conversely, the low fertility but deep and freely-draining soils of the eastern Amazon tend to have lower productivity and slower turnover, and species with much higher wood density. The relationship between diameter and height also varies across the basin, but with more complexity than wood density, mostly related to climatic factors (Feldpausch *et al*., 2011). These are approximated into four zones (Feldpausch *et al*., 2011, 2012), with the use of a different *D*:*H* model in each zone, greatly reducing the error in the prediction of *H* from *D* compared to a pan-Amazonian model (Feldpausch *et al*., 2012).

Thus, although *D*, ρ and *H* were used in the field-plot calibration of *RS1*, and *D* and ρ for *RS2* (with the allometric equations used coming from the same study: Chave *et al*., 2005), regional differences in *D:H* ratios and ρ are not detected by GLAS, and thus the continental-scale GLAS–AGB calibrations used could smooth out these differences. This is likely to result in significantly higher regional uncertainties than estimated by Saatchi *et al*. (2011) or Baccini *et al*. (2012).

**Figure 1:**
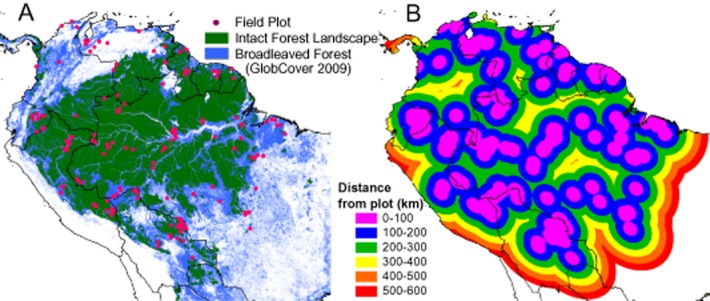
Location of forest field plots in South America. (a) The location of all plots used in the analysis, overlaid on the intact forest landscape (IFL) and GlobCover broad-leaved forests layers. (b) Map showing the distance from the nearest plot in kilometres.

In order to test this, we use a unique dataset of 413 field plots located throughout tropical South America, compiled as part of RAINFOR (*Red Amazónica de Inventarios Forestales*; Amazon Forest Inventory Network; Malhi *et al*., 2002), the Amazon Tree Diversity Network (ter Steege *et al*., 2003), TEAM (Tropical Ecology Assessment and Monitoring) and PPBio (Brazilian Program for Biodiversity Research) (Fig. 1). Data in these plots were collected using a consistent methodology, and AGB was calculated using a T-SQL query to a single database. We compare these field plots directly to the two remote-sensing-derived maps, and additionally create a plot-based AGB map using simple two-dimensional kriging (*K*_*DH*ρ_) to allow a spatial comparison.

## Materials and Methods

Details of field methods and error checking procedures involved in the RAINFOR permanent plot network are discussed in detail elsewhere (Phillips *et al*., 2008, 2009a, b). The individual stem data for every plot used in this study are held in a database (http://www.forestplots.net/), which allowed us to calculate plot-level AGB consistently with a single T-SQL query (Lopez-Gonzalez *et al*., 2009, 2011). The TEAM plots were downloaded and added to the database in April 2013, with data set identifier codes of 20130415013221_3991 and 20130405063033_1587. We only used plots where data were available for every stem and trees had been measured consistently above buttresses. Plots above 1000 m elevation were excluded, as were plots in non-forest ecosystems. On average across plots, 77% of stems were identified to the species level, and 92% to the genus level. The dates at which the plots were most recently measured, and the number of times they had been re-censused, varied: in order to dampen the influence of short-term disturbances and to produce values that most closely represented the landscape AGB distribution, the value for each plot was calculated as the mean of all census values, weighted by census interval lengths before and after each measurement. Censuses collected from 2010 onwards were excluded as these post-date the remote-sensing data, apart from 41 plots that were only measured for the first time during or after 2010, in which case the earliest available census was used.

The principal AGB dataset was calculated using the three-parameter moist tropical forest model from Chave *et al*. (2005), with height estimated from d.b.h. individually for each stem using the region-specific Weibull models from Feldpausch *et al*. (2012), and wood density values estimated for each stem using the mean value for the species in the Global Wood Density Database (Chave *et al*., 2009; Zanne *et al*., 2009), or the mean for the genus using congeneric taxa from Mexico, Central America and tropical South America if no data were available for that species (*K*_*DH*ρ_). For comparison, AGB was also calculated using the same allometric equation but with the pan-Amazon Weibull model from Feldpausch *et al*. (2012) (*K*_*D*ρ_), regional height models but with a dataset mean wood density value of 0.63 applied to every stem (*K_DH_*), and with the pan-Amazonian height model and mean wood density applied to every stem (*K_D_*).

In order to compare the AGB dataset directly with the field plots, we averaged the field plots within 20 km × 20 km boxes and compared the mean value for these boxes to the mean AGB of *RS1* and *RS2.* This was intended to reduce the noise involved in comparing single field plots to their surrounding remote-sensing pixel. This resulted in comparisons being made with 107 unique points, with a mean of 3.9 field plots in each (range 1–14).

We attempted to produce the kriged maps using universal kriging (ter Steege *et al*., 2003), but this proved impossible because of high local variation in AGB values of neighbouring plots, resulting in little spatial autocorrelation. Plots located within a 250-m search radius were averaged, which reduced the total number of independent points entering the kriging procedure from 413 to 378; this assisted matters, but a semivariogram showed that there was still little spatial autocorrelation in the dataset (Fig. S1). We therefore used an inverse distance kernel with a smoothing distance of 100 km, which removed local variation and produced output layers showing the broad spatial trends in the dataset. The output kriged maps were produced at a 500-m resolution using the MODIS sinusoidal projection, an equal-area projection used in the creation of *RS2.*

*RS1* was provided by S. Saatchi (NASA Jet Propulsion Laboratory, CA, USA) in a geographic projection with a pixel size of 0.00833°; *RS2* was provided by A. Baccini (Woods Hole Research Center, MA, USA) in a MODIS sinusoidal projection at 500 m resolution. *RS1* was warped to the projection of *RS2* using an exact mathematical transformation. Pixel values were assigned during warping using the ‘nearest neighbour’ algorithm, so no pixel values were changed by the warping procedure.

The units of the maps were in tonnes of biomass per hectare (Mg ha^−1^). Total carbon stocks for subsets of the resulting layers were calculated by multiplying the mean biomass of a subset by its area in hectares, and then converting biomass to carbon by multiplying the result by 0.5 (as dry biomass is assumed to be 50% carbon; Penman *et al*., 2003).

All the plots entering the kriged map were located in forest areas with no recent anthropogenic disturbance, but a significant proportion of Amazonia is non-forest or degraded forest (Fig. 1a). Unsurprisingly, *K*_*DH*ρ_ overpredicted AGB in all areas dominated by non-forest land-cover types compared to the RS maps. Therefore the maps are most comparable in undisturbed forest areas, so all comparisons were performed in Intact Forest Landscape (IFL) (Potapov *et al*., 2008) areas only, with the exception of the analysis of recent deforestation. IFLs are defined as forest areas minimally influenced by human economic activity, with an area of at least 50,000 ha and a minimum width of 10 km. The IFL layers are kept updated for new infrastructure, settlements or commercial activities by their developers using a combination of field data and remote-sensing data (Potapov *et al*., 2008).

## Results and Discussion

**Figure 2:**
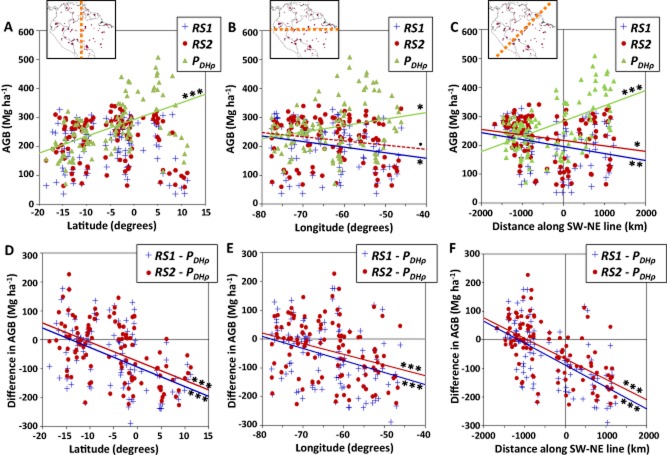
Best estimates field plot above-ground biomass (AGB) in the Amazon forests (*P*_*DH*ρ_), averaged in 20 km × 20 km boxes (*n* = 107), and AGB derived from two remote-sensing-derived maps, *RS1* (Saatchi *et al*., 2011) and *RS2* (Baccini *et al*., 2012) for the same boxes, plotted against (a) latitude, (b) longitude and (c) distance along a SW–NE (45° bearing) line, centred on Manaus; and difference between the RS layers and *P*_*DH*ρ_ plotted against (d) latitude, (e) longitude and (f) distance along a SW–NE line. Best-fit lines are shown if significant; · *P* < 0.1; * *P* < 0.05; ** *P* < 0.01, *** *P* < 0.001.

**Figure 3:**
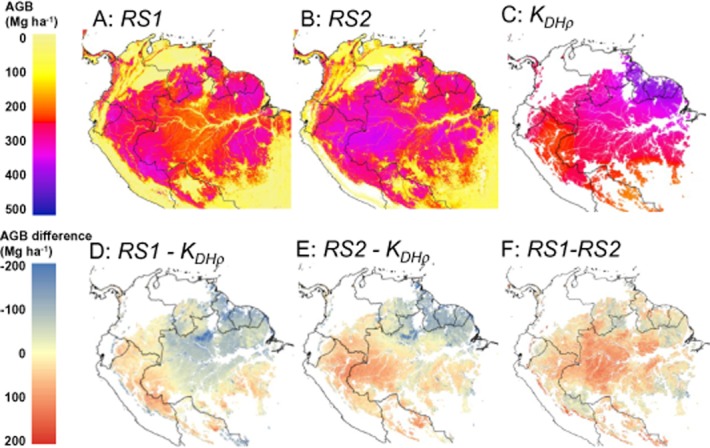
Above-ground biomass (AGB) maps of South America and maps of their differences. (a) AGB map *RS1* (Saatchi *et al*., 2011); (b) AGB map *RS2* (Baccini *et al*., 2012); (c) kriged map of AGB from field plots, with AGB calculated using diameter, species-specific wood density, and a regional height model (*K*_*DH*ρ_), showing only areas identified as intact forest landscapes (IFL); (d) difference between *RS1* and *K*_*DH*ρ_; (e) difference between *RS2* and *K*_*DH*ρ_; (f) difference between *RS1* and *RS2.* Difference maps have non-IFL areas masked out. The projection is sinusoidal, an equal-area projection.

The field plots with our best estimate of AGB (*P*_*DH*ρ_) show a robust trend of increasing AGB with increasing latitude, longitude and distance along a SW–NE line (Fig. 2a–c; the parameters of the best-fit lines are given in Table S1; input plot biomass data are available in Lopez-Gonzalez *et al*., 2014). The kriged map of the same field plots (Fig. 3c) shows that the latitudinal and longitudinal trends seen in the graphs are clearly driven by the dominant SW–NE gradient. By contrast, the remote-sensing layers *RS1* and *RS2* show significant decreasing trends with distance along a SW–NE line (Fig. 2c, Table S1). Subtracting the two RS layers from the plot AGB values emphasizes the trends described above, with positive differences (i.e. *RS1* and *RS2* greater than *P*_*DH*ρ_) in the south and west, and negative differences in the north and east (Fig. 2d–f, Table S1).

Two-dimensional kriging of the plot dataset (*K*_*DH*ρ_) allows area-based comparisons to be made between the field-plot and RS datasets. The total Amazon basin AGB stocks in *RS1*, *RS2* and *K*_*DH*ρ_ did not differ greatly for intact forest landscape (IFL) areas (Potapov *et al*., 2008) (Table 1), with *K*_*DH*ρ_ and *RS2* having very similar total stocks, and the *RS1* estimate being 11% lower. The patterns of AGB differ greatly among the maps, however, as shown in Fig. 3 and demonstrated by the high root mean squared error (RMSE) values comparing *RS1* and *RS2* to *K*_*DH*ρ_ (Table 1). The two RS maps do not show the strong SW–NE AGB gradient seen in *K*_*DH*ρ_ (Figs 2 & 3). *RS1* shows similar AGB in western and eastern Amazon forests, with distinctly lower AGB in central Amazonia than either of the other maps, explaining its lower total stock estimate. *RS2* has less variation in AGB overall, but with the highest values in the central-western Amazon, opposite to the pattern seen in the *K*_*DH*ρ_ map.

**Table 1:** Comparison of the mean above-ground biomass (AGB) and total above-ground carbon stock contained in two remote-sensing-derived maps of the Amazon forests (*RS1*, Saatchi *et al*., 2011; *RS2*, Baccini *et al*., 2012) with a map derived from kriging 413 field plots (*K_DH_*_ρ_), and maps derived from these same field plots but excluding wood density, local tree height allometry, or both (*K_DH_*, *K_D_*_ρ_ and *K_D_*, respectively). In all cases, only intact forests are considered (Potapov *et al*., 2008). RMSE, root mean squared error, is calculated on a 500 m pixel basis

Map	Mean AGB (Mg ha^−1^)	Total carbon stock (Pg C)	% difference from *K_DH_*_ρ_	RMSE from *K_DH_*_ρ_ (Mg ha^−1^)
Amazonia (423,869,500 ha)				
* K_DH_*_ρ_	287.0	60.83	n/a	n/a
* RS1*	255.0	54.05	−11.1%	83.4
* RS2*	285.5	60.52	−0.5%	77.1
* K_DH_*	278.6	59.04	−2.9%	19.3
* K_D_*_ρ_	281.8	59.72	−1.8%	40.5
* K_D_*	275.6	58.41	−4.0%	45.3
NE Guiana Shield* (32,065,200 ha)				
* K_DH_*_ρ_	387.9	6.22	n/a	n/a
* RS1*	279.5	4.48	−27.9%	123.6
* RS2*	278.8	4.47	−28.1%	117.4
* K_DH_*	355.0	5.69	−8.5%	33.7
* K_D_*_ρ_	350.3	5.62	−9.7%	38.1
* K_D_*	321.3	5.15	−17.2%	67.3
SW Amazonia† (43,155,200 ha)				
* K_DH_*_ρ_	244.3	5.27	n/a	n/a
* RS1*	283.2	6.11	15.9%	66.4
* RS2*	290.5	6.27	18.9%	64.6
* K_DH_*	266.4	5.75	9.1%	22.8
* K_D_*_ρ_	251.6	5.43	3.0%	7.7
* K_D_*	274.8	5.93	12.5%	31.2

* Guyana, Suriname & French Guiana.

† Acre Basin, Beni Basin, Madre de Dios Basin, Ucayali Basin.

We believe that the AGB gradient in the field plots cannot be an artefact of our analysis: the ground-based estimates use consistent field measurements (Phillips *et al*., 2008, 2009a) in well-surveyed plots, with AGB calculated at a stem level using a trusted allometric equation (Chave *et al*., 2005) involving tree diameter, wood density and height. We are confident that diameters are measured to high precision, as a primary purpose of these plots is to track small changes in diameter through time; we are confident that our wood density values are accurate due to careful species identification and the use of a reliable wood-density dataset (Chave *et al*., 2009; Zanne *et al*., 2009); and we account for spatial variation in stem height (Feldpausch *et al*., 2011). While *K*_*DH*ρ_ is clearly not an accurate spatial map, being based solely on 413 point measurements, these are well distributed across the study area and thus should correctly display broad regional trends in forest structure: every IFL pixel in Amazonia is within 500 km of at least eight plots (Fig. 1b). A semivariogram analysis of the field data shows that the spatial autocorrelation of plots does not start to decrease until they are about 700 km apart, suggesting this plot network is sufficient to represent the potential AGB of old-growth forests across the whole basin (Fig. S1).

It is clear that the use of *RS1* or *RS2* for carbon accounting purposes for a subset of this area will produce very different results from those using *K*_*DH*ρ_: the RS maps will underestimate stocks in the Guiana Shield and overestimate in SW Amazonia (Table 1). To demonstrate the difference for a relevant example, we calculated the estimated emissions from deforestation in Brazil from 2009–2011, after the reported date of either RS map, using the PRODES dataset (INPE, 2012). We found significantly higher carbon estimates with *K*_*DH*ρ_ than with either RS map (Table 2). It is possible that estimates from the RS maps are lower because these areas were already degraded at the time the maps were made, with this perhaps explaining the lower value for *RS2* than *RS1*, as *RS2* is produced for a date *c.* 5 years later than *RS1*. However, this is unlikely to be the case in all areas, and thus cannot explain the extent of the difference.

**Table 2:** Above-ground biomass (AGB) contained in areas deforested between 2009 and 2011 in Brazil using the PRODES dataset (INPE, 2012). The total area deforested was 1,853,610 ha

Map	Mean AGB (Mg ha^−1^)	Total carbon stock (Tg C)	% difference from *K_DH_*_ρ_
*K_DH_*_ρ_	275.7	511.0	
RS1	206.4	382.6	−25.1%
RS2	176.6	327.4	−35.9%

**Figure 4:**
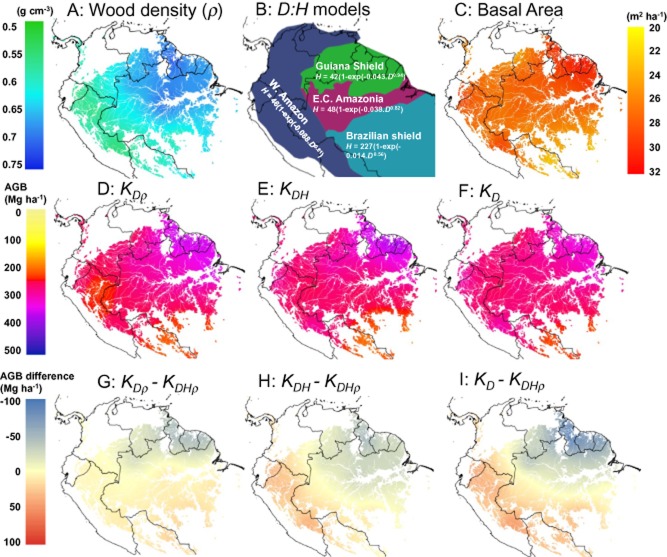
Drivers of the above-ground biomass (AGB) distribution seen in field plots in South America. (a) Kriged map of mean wood density (*ρ*) (mean calculated using basal-area weighting when summing stems). (b) Map showing the regions of differing tree diameter (*D*) to tree height (*H*) equations developed in (Feldpausch *et al*., 2012) used for estimating *H* from *D* in *K_DHρ_* and *K_DH_* (c) Kriged map of basal area. (d) Kriged map of AGB using *D* and species-specific *ρ*, but a pan Amazonian height model (*K_Dρ_*). (e) Kriged map of AGB using *D*, regional height models and *ρ*, but with *ρ* fixed at 0.63 (*K_DH_*). (f) Kriged map of AGB using *D*, pan-Amazonian height model, and *ρ* fixed at 0.63 (*K_D_*). (g–i) Differences between the named kriged map layers.

It is important to understand the drivers of the AGB gradient seen in the field plots. From an ecological point of view, AGB is ultimately a function of net primary production (NPP) and the turnover rate of the forest. Spatial differences in NPP and turnover rates are associated with different species with different life-history strategies and structures caused by different climatic conditions and the chemical and physical properties of the soil (Quesada *et al*., 2012), with these different floristic communities associated with different AGB values. The key ecological parameters associated with differing AGB are basal area, wood density and *D*:*H* ratios, all of which vary across the basin (Baker *et al*., 2004; ter Steege *et al*., 2006; Banin *et al*., 2012; Feldpausch *et al*., 2012; Quesada *et al*., 2012), but none of which is directly detected by RS. Mapping basal area and wood density from the plots shows that both increase from SW–NE, though wood density shows a larger proportional trend than basal area (Fig. 4a,c). It is also known that, in general, tropical South American trees are shorter for a given diameter than trees from other tropical regions (Banin *et al*., 2012), with the exception of the Guiana Shield where trees are comparatively tall for a given diameter, with *D*:*H* relationships statistically indistinguishable from the forests of Africa and Southeast Asia (Feldpausch *et al*., 2011). In order to assess the relative impact of wood density and tree height on AGB, we recalculated the AGB values of the 413 field plots using the same three-parameter allometric equations and diameter information, but applying three different approaches to the other two variables:

1. *K*_*D*ρ_: Using a pan-Amazonian *D*:*H* model rather than the four regional height models (Feldpausch *et al*., 2012), but the same wood density values as *K*_*DH*ρ_;
2. *K*_*DH*_: Using a constant value of 0.63 as the wood density for every stem, but the regional height models as in *K*_*DH*ρ_;
3. *K*_*D*_: Using a pan-Amazonian *D*:*H* model and a constant wood density value, but the same allometric equations, so AGB varied between plots solely due to *D*.

*K*_*D*ρ_ and *K_DH_* both have significant SW–NE AGB gradients, albeit less marked than for *K*_*DH*ρ_ (Fig. 4)_._ On average across the Amazon, the exclusion of wood density (*K_DH_*) leads to a small reduction in predicted AGB compared to *K*_*DH*ρ_, but this reduction is bigger when considering only the Guiana Shield (known to have high wood density), and is reversed in SW Amazonia (known to have lower woody density) (Fig. 4, Table 1). Using a pan-Amazonian height model (*K*_*D*ρ_) leads to a very small reduction in overall predicted AGB across the Amazon, with a decrease in the Guiana Shield, and a matching increase in SW Amazonia. Excluding both height and wood density (*K_D_*) again results in an small reduction in predicted AGB for the whole basin, with a very large underestimate in the Guiana Shield (−17.2%), and a smaller overestimate in SW Amazonia (12.5%) (Fig. 4, Table 1). From this analysis, we conclude that the RS layers underestimate AGB in the Guiana Shield due to a contribution of using mean wood density (an underestimate) and a generic pan-Amazonian relationship between diameter and height (ignoring the fact that trees are taller than would be expected for a given diameter in this region), with the two factors having approximately equal contributions. In SW Amazonia, the difference is caused by the same two factors in the opposite direction: using pan-Amazonian wood density and *D:H* relationships here results in an overestimate of AGB. In SW Amazonia the two factors are not equal in magnitude, with wood density causing approximately three times more overestimation than the *D*:*H* relationship (Table 1).

Wood density and *D*:*H* relationships alone, however, cannot explain all the differences between *RS1/RS2* and *K*_*DH*ρ_. In both the Guiana Shield and the SW Amazon, the difference between *K*_*DH*ρ_ and *K_D_* is smaller than the difference between *K*_*DH*ρ_ and the RS maps. The unexplained difference is over 10% for the Guiana Shield, and 3–5% for the SW Amazon. There must therefore either be further factors in the processing chains involved in developing *RS1* and *RS2* from their input datasets that contribute to the over- and under-estimation in these regions, or the non-random nature of the input field datasets must be causing this additional difference. We believe the latter explanation is unlikely, as any bias towards pristine forests would tend to cause an overestimate in the plot-based estimate, but instead the field plots estimate lower AGB in SW Amazonia. Possible explanations for the former include incorrect or saturating relationships between GLAS footprints and AGB, or explanatory variables not fully capturing the spatial variability in forest structure, causing the resulting maps to tend towards the mean. The differences between *RS1* and *RS2* themselves are caused in part by the use of different remote-sensing datasets, different methods of processing the GLAS data, and different extrapolation approaches, but also by the choice of allometric equation to convert their field plot data into AGB estimates. *RS2* uses a two-parameter equation excluding height, whereas *RS1* uses the three-parameter equation we used to estimate AGB from our plots, albeit with effectively a pan-Amazonian *D*:*H* relationship. The equation excluding height used in *RS2* is known to estimate higher AGB values than the three-parameter equation used in *RS1* (Chave *et al*., 2005; Feldpausch *et al*., 2012), so this choice probably explains much of the 12% higher total AGB estimate for Amazonia by *RS2* compared to *RS1* (Table 1).

## Conclusions

The two remote-sensing maps, *RS1* (Saatchi *et al*., 2011) and *RS2* (Baccini *et al*., 2012), show very different spatial patterns of AGB distribution across Amazonia, compared to each other and compared to field plots distributed across the region (Figs 2 & 3). In particular, the strong gradient of increasing AGB from SW to NE Amazonia that we observe in the field data is not replicated in the RS datasets. *RS1* and *RS2* do have associated uncertainty estimates, but the differences observed between the maps and field plots considerably exceed the reported uncertainties over Amazonia in both cases. Our analysis shows that this is mostly due to neither study accounting for the known regional variations in wood density and *D*:*H* relationships. Specifically, they do not use any spatial layers corresponding to wood density, and use only continental (Saatchi *et al*., 2011) or global (Baccini *et al*., 2012) relationships between ICESat GLAS waveforms and AGB.

We are not advocating the use of extrapolated AGB maps derived from sparse field measurements for carbon accounting: we firmly believe that AGB is best mapped using a combination of RS data calibrated and validated using a substantial number of carefully established field plots. It is this step of careful validation against best estimates from scientific field plots that we believe was lacking in the RS studies. Looking forward, we provide the following recommendations to improve AGB estimates. First, regional differences in wood density and *D*:*H* relationships must be considered when mapping AGB. One universal algorithm predicting AGB from a suite of remote-sensing variables is not appropriate, as wood density cannot be detected from space, and the structural parameters of forests cannot yet be reliably extracted from RS data. Different algorithms should be applied to different regions, potentially based on maps of soil type or vegetation structure. Alternatively spatially explicit maps of wood density and diameter:height relationships should be directly incorporated into biomass mapping algorithms (Asner *et al*., 2012). There is a clear need for the ecological community to provide regional and pantropical maps of basal-area-weighted wood density based on plot data, potentially extrapolated using climate or other layers, as inputs for the biomass mapping communities. Second, our study demonstrates the importance of creating and sustaining large networks of field plots. This analysis was only possible because a sufficient number of plots have been located across the basin using a standard methodology, with the data included in a single database allowing identical processing chains to be applied to stem data. It is important that such networks are maintained and improved across the tropics, and vital that current spatial data gaps are filled (Fig. 1b). Third, measuring tree height and identifying the thousands of Amazon tree species (at least to genus level, enabling stems to be matched to wood density information), were essential components of the field data. Recording tree diameters alone would not have allowed us to identify these important regional gradients in AGB: variation in biodiversity can matter greatly for determining carbon stocks.

## Supporting Information

**Figure S1** Semivariogram showing how variance between biomass values for the field plots varies with distance. **Table S1** Parameters for the fits in Figure 2. The permanent archive of the field plot data can be accessed at: http://dx.doi.org/10.5521/FORESTPLOTS.NET/2014_1
