# Supplementary material for: Markedly divergent estimates of Amazon forest carbon density from ground plots and satellites
Source: Glob Ecol Biogeogr. 2014 Apr 22;23(8):935–46. doi: 10.1111/geb.12168 (PMC4579864; doi:10.1111/geb.12168)
Supplement: Supplementary file 1 — Figure S1 Semivariogram showing how variance between biomass values for the field plots varies with distance. Table S1 Parameters for the fits in Figure 2. The permanent archive of the field plot data can be accessed at: http://dx.doi.org/10.5521/FORESTPLOTS.NET/2014_1 [file geb0023-0935-sd1.docx]

*Global Ecology and Biogeography*

**Supporting Information**

**Markedly divergent estimates of Amazon forest carbon density
from ground plots and satellites**

Edward T. A. Mitchard, Ted R. Feldpausch, Roel J. W. Brienen *et al.*


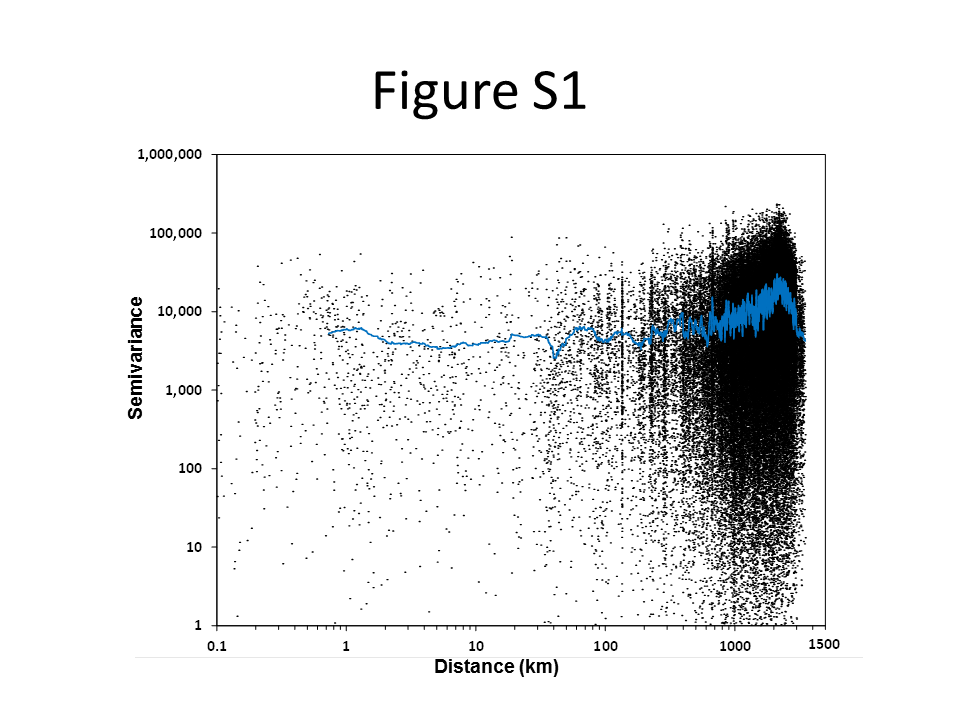


**Figure S1** Semivariogram showing how the variance between pairs of aboveground biomass values from 413 large field plots from the Amazon basin varies with distance. The blue line represents a moving average of 100 points.

**Table** **S1** Statistics describing the best-fit lines shown in Fig. 2. Significance: n.s., *P* > 0.05; •, *P* < 0.1; **P* < 0.05; ***P* < 0.01; ****P* < 0.001.

| Regression | *a* | *b* | Adjusted *r*^2^ | Regression *F*-values (*F*_1,105_) | *P* | Significance |
| --- | --- | --- | --- | --- | --- | --- |
| RS1 vs. latitude | 199.2 | −0.955 | −0.001 | 0.94 | 0.3354 | n.s. |
| RS2 vs. latitude | 219.5 | −0.814 | −0.003 | 0.69 | 0.4093 | n.s. |
| *P_DH_*_ρ_ vs. latitude | 293.4 | 5.801 | 0.248 | 35.95 | 2.90 × 10^−8^ | *** |
| RS1-*P_DH_*_ρ_ vs. latitude | −94.2 | −6.756 | 0.232 | 32.96 | 9.21 × 10^−8^ | *** |
| RS2-*P_DH_*_ρ_ vs. latitude | −73.9 | −6.615 | 0.233 | 33.19 | 8.43 × 10^−8^ | *** |
| RS1 vs. longitude | 81.5 | −1.930 | 0.039 | 5.34 | 0.0228 | * |
| RS2 vs. longitude | 131.8 | −1.448 | 0.018 | 2.97 | 0.0877 | • |
| *P_DH_*_ρ_ vs. longitude | 406.7 | 2.252 | 0.042 | 5.70 | 0.0188 | * |
| RS1-*P_DH_*_ρ_ vs. longitude | −275.0 | −3.700 | 0.114 | 14.67 | 0.0002 | *** |
| RS2-*P_DH_*_ρ_ vs. longitude | −325.2 | −4.182 | 0.092 | 11.75 | 0.0009 | *** |
| RS1 vs. SW–NE line | 195.2 | −0.024 | 0.059 | 7.69 | 0.0066 | ** |
| RS2 vs. SW–NE line | 216.7 | −0.019 | 0.034 | 4.72 | 0.0321 | * |
| *P_DH_*_ρ_ vs. SW–NE line | 283.3 | 0.053 | 0.249 | 36.14 | 2.70 × 10^−8^ | *** |
| RS1-*P_DH_*_ρ_ vs. SW–NE line | −88.1 | −0.077 | 0.369 | 63.02 | 2.38 × 10^−12^ | *** |
| RS2-*P_DH_*_ρ_ vs. SW–NE line | −66.6 | −0.072 | 0.337 | 54.86 | 3.40 × 10^−11^ | *** |
